# Urgent or Emergent Endovascular Aortic Repair of Infective Aortitis

**DOI:** 10.3390/jcm13164669

**Published:** 2024-08-09

**Authors:** Bernardo Orellana Davila, Carlotta Mancusi, Carlo Coscarella, Claudio Spataro, Paolo Carfagna, Arnaldo Ippoliti, Rocco Giudice, Ciro Ferrer

**Affiliations:** 1 Vascular and Endovascular Surgery Unit, San Giovanni-Addolorata Hospital, 00184 Rome, Italy; bernardo.orellana.d@gmail.com (B.O.D.); carlotta.mancusi@gmail.com (C.M.); carlo.coscarella@gmail.com (C.C.); cspataro86@gmail.com (C.S.); rgiudice@hsangiovanni.roma.it (R.G.); 2 Clinical Medicine and Infectious Diseases Unit, San Giovanni-Addolorata Hospital, 00184 Rome, Italy; pcarfagna@hsangiovanni.roma.it; 3 Vascular Surgery Unit, Biomedicine and Prevention Department, University of Rome Tor Vergata, 00133 Rome, Italy; ippoliti@med.uniroma2.it

**Keywords:** aortitis, abdominal aortic aneurysm, thoracic aortic aneurysm, thoracoabdominal aortic aneurysm, endovascular aneurysm repair, aortic infection, aortic inflammation

## Abstract

**Background:** Aortitis is a rare inflammation of the aorta. It can be classified as infective, non-infective, or idiopathic. Infective aortitis can debut as an acute aortic syndrome that needs urgent or emergent treatment. Historically, these kinds of patients have been preferably treated by open surgery, with subsequent lack of information about the endovascular repair. The aim of the present study is to report the results of our experience with the urgent or emergent endovascular repair of infective aortitis with acute presentation. **Methods:** All consecutive urgent or emergent endovascular repairs, performed between January 2019 and January 2024 for the treatment of infective aortitis, were included. The inclusion criteria were clinical, laboratory, and radiological findings recognized as aortitis risk factors. Patients with graft or endograft infection, aortic fistulae, and mycotic aneurysm were excluded. Primary endpoints were technical success and 30-day and follow-up survival. Early and late major adverse events, any changes in lesion morphology over time, and need for re-intervention were also assessed. **Results:** A total of 15 patients (14 males and 1 female) with a mean age of 74.2 ± 8.3 were included. All the subjects were treated by endovascular means in an urgent or emergent setting because of a rapidly growing aneurysm, symptomatic lesion, or contained or free aortic rupture. The diagnosis of infective aortitis was confirmed postoperatively by positive blood cultures in all the patients. A rapidly growing or symptomatic lesion was noted in all 15 subjects. Among these there were six (40%) contained and two (13%) free aneurysm ruptures. The endovascular techniques performed were as follows: four thoracic-EVAR (TEVAR), three off-the-shelf branched-EVAR (BEVAR), one Chimney-EVAR (Ch-EVAR), six EVAR with bifurcated graft, and one EVAR with straight tube graft. Technical success was achieved in 100% of the patients. Two patients (13%) died within 30 days after the index procedure. No case of early aortic-related mortality was registered. During a mean follow-up of 31.6 ± 23.1 months (range 1–71), no further death or major adverse event was registered among the remaining 13 alive patients. Re-interventions were performed in three cases (20%). Aneurysm’s shrinkage > 5 mm or stability was noted in 10 of the 13 patients who survived the early period after repair. **Conclusions:** Despite the relative reluctance to use an endograft in an infected area, in our experience the endovascular approach resulted to be feasible, safe, and effective in the treatment of infective aortitis with acute presentation, with acceptable peri-operative and mid-term follow-up outcomes. Further studies are needed to confirm our results.

## 1. Introduction

Aortitis is an inflammation of the aorta. It can be infective, non-infective, or idiopathic. The most common causes of non-infective aortitis are large vessel vasculitis and giant cell and Takayasu arteritis, although it can be associated with other rheumatologic diseases. While the majority of cases of aortitis are non-infective, the possibility of an infective cause should always be considered, as treatment strategies may widely vary. Infective aortitis can originate from the bloodstream, septic embolus, or infected surrounding tissues [1]. A number of organisms have been associated with infective aortitis, most commonly Salmonella and Staphylococcal species [2]. Occasionally, an infective aortitis may occur as a result of direct seeding of the aorta from adjacent infected tissues, such as in case of tuberculosis, pancreatitis, and spondylodiscitis. In most cases of infective aortitis, a segment of the aortic wall with pre-existing pathology, such as an atherosclerotic plaque, porcelain aorta, or aneurysm sac, is seeded by bacteria via the vasa vasorum. Indeed, a healthy aorta is generally resistant to inflammatory insults, but in the event of severe atherosclerosis, its wall may be more prone to inflammation.

The process is based on a chronic inflammatory infiltration of the medial and adventitial vasa vasorum, which ultimately leads to a medial necrosis. The diagnosis of aortitis is challenging due to the lack of specificity of the symptoms and the variable clinical picture: not-well-defined thoracic or abdominal pain, fever of unknown origin, and signs of sepsis. In some cases, aortitis results to be an incidental finding at the time of histopathologic examination following surgery for aortic aneurysm [3].

Infective and non-infective aortitis are both potentially life-threatening conditions [4]. Indeed, regardless of its etiology, aortitis can debut as an acute aortic syndrome that needs urgent or emergent treatment. Historically, these kinds of patients have been preferably treated by open surgery. Therefore, the reluctance to insert a stent graft into an infected tissue has led to the lack of information about the endovascular approach in this scenario, limiting the data to case reports or very small case series. However, the increasing experience and technical skills acquired by many vascular centers, together with the availability of the latest generation of devices for endovascular aneurysm repair (EVAR), has also made such an approach more and more popular for this spectrum of diseases, especially in emergent situations [5]. In this scenario, the aim of the present study is to report the results of our experience with the urgent or emergent endovascular repair of infective aortitis with acute presentation.

## 2. Methods

### 2.1. Study Design

Data regarding patients who underwent urgent or emergent endovascular aortic repair from two tertiary vascular centers were prospectively collected between January 2019 to January 2024 and retrospectively reviewed. As per our practice, all patients presenting with ruptured, symptomatic, or rapidly growing aortic aneurysm were investigated with the purpose of identifying any potential infective etiology. The study was performed in accordance with the Institutional Ethical Committee rules, and individual consent for this retrospective analysis was waived. All patients provided consent for intervention.

### 2.2. Inclusion and Exclusion Criteria

Clinical risk factors taken into account were as follows: current or previous sepsis, endocarditis, pneumonia, pancreatitis, and spondylodiscitis. Some peculiar radiological findings, such as saccular, multi-lobular, or eccentric lesions, pseudo-aneurysmal morphology, peri-aortic thickening > 4 mm, peri-aortic effusion, and peri-aortic gas, were also assessed. The diagnosis of aortitis was based on the presence of one or more of the abovementioned criteria and was always confirmed by positive blood cultures. Patients with graft or endograft infection, aortic fistulae (bronchial, esophageal, or enteric), and mycotic aneurysm were excluded. Given their rarity in our experience, elective procedures in patients with suspected aortitis were also excluded.

### 2.3. Preoperative and Postoperative Management

Multiplanar reconstructions of preoperative computed tomography angiography (CTA) according to the centerline of flow were analyzed using Aquarius iNtuition vers. 4.4.13.P5 (TeraRecon, Foster City, CA, USA) with the purpose of evaluating the feasibility of the endovascular approach and choosing the most suitable stent graft configuration. Endovascular devices included standard abdominal or thoracic grafts and off-the-shelf branched endoprosthesis, according to the disease’s extension. Endovascular procedures with parallel endografts such as chimney or periscope techniques were occasionally performed based on the patient’s needs.

Postoperatively, a follow-up scheme was devised based on clinical and CTA surveillance at 1, 6, and 12 months, followed by annual control visits thereafter. In most of the cases, the first postoperative CTA was performed before discharge. Postoperative drug therapy included targeted antibiotic therapy established as soon as the results of blood cultures were available. Inflammatory status was postoperatively monitored for each patient by conducting serial blood tests for the value of white blood cells (WBC), C-reactive protein (CRP), erythrocyte sedimentation rate (ESR), and procalcitonin (PCT). An infectious disease specialist was always consulted for the interpretation of laboratory findings and the management of antibiotic therapy.

### 2.4. Endpoints Definition

Primary endpoints included technical success and 30-day and follow-up survival. Early and late major adverse events, any changes in lesion morphology over time, and the need for re-intervention were also assessed. Technical success was defined as correct delivery and deployment at the intended location of all the endografts, with patency of the grafts and any aortic branch incorporated, and without any high-flow angiographically detected endoleak.

### 2.5. Statistical Analysis

Measured values are reported as percentages and means ± standard deviation or medians and interquartile ranges. Comparative data are expressed as odds ratios and corresponding 95% confidence intervals. A *p* value of less than 0.05 was considered statistically significant.

## 3. Results

### 3.1. Population

A total of 15 patients (14 males and 1 female) with a mean age of 74.2 ± 8.3 (range 61–87) were included. All the subjects were treated by endovascular means in an urgent or emergent setting because of a rapidly growing aneurysm, symptomatic lesion, or contained or free aortic rupture. The diagnosis of infective aortitis was confirmed postoperatively by positive blood cultures in all the patients. Organisms associated with infective aortitis were as follows: *Staphylococcus aureus* (7), *Salmonella* spp. Non-Typhi (3), *Escherichia coli* (3), *Brucella* spp. (1), and association of *Pseudomonas* and *Klebsiella* (1). Patients’ medical history included the following: sepsis (3), spondylodiscitis (3), endocarditis (4), pancreatitis (2), pyelonephritis (2), and pneumonia (1). All patients presented neutrophilia (mean value: 84.32% ± 6.88), an elevated level of C-Reactive-Protein (mean value: 38.50 ± 91 mg/dL), and a significant aortic wall thickening (32.5 ± 20.26 mm). A rapidly growing or symptomatic lesion was noted in all 15 subjects. Among these there were six (40%) contained and two (13%) free aneurysm ruptures. The endovascular techniques performed were as follows: four thoracic-EVAR (TEVAR), three off-the-shelf branched-EVAR (BEVAR), one Chimney-EVAR (Ch-EVAR), 6 EVAR with bifurcated graft, and one EVAR with straight tube graft. The baseline characteristics of the patients are reported in Table 1.

### 3.2. Early Results

The mean in-hospital stay was 13.5 ± 11.0 days. All the patients were admitted to the intensive care unit for a mean of 3.2 ± 2.1 days. Technical success was achieved in 100% of the patients. Two patients (13%) died within 30 days after the index procedure. The causes of the two early deaths were multi-organ failure likely secondary to sepsis in a patient with contained ruptured saccular thoracic aneurysm, and myocardial infarction in a patient presenting with ruptured infrarenal aneurysm. No case of early aortic-related mortality was registered. Two patients (13%) required temporary dialysis after repair. All patients were discharged with long-term targeted antibiotic therapy administered for a mean of 51 ± 28.6 days.

### 3.3. Late Results

**Figure 1:**
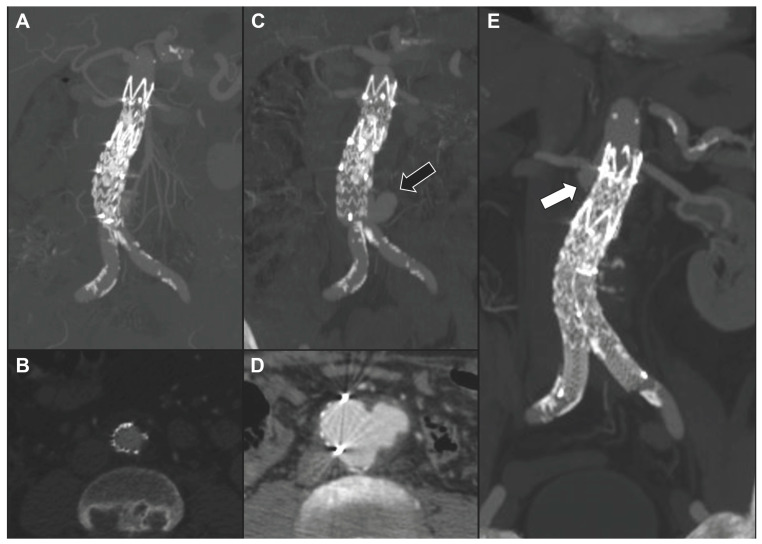
One-month computed tomography angiography (CTA) showing a good result after straight EVAR (with 2 cuffs) for contained ruptured infrarenal atheromatous plaque in maximum intensity projection (**A**) and axial view (**B**). The same patient experienced, after 5 months, distal disease progression with rupture of the aortic bifurcation (black arrow) (**C**,**D**). The 55-month CTA showed the exclusion of the infrarenal lesion with initial degeneration of the proximal aortic neck (white arrow) (**E**).

**Figure 2:**
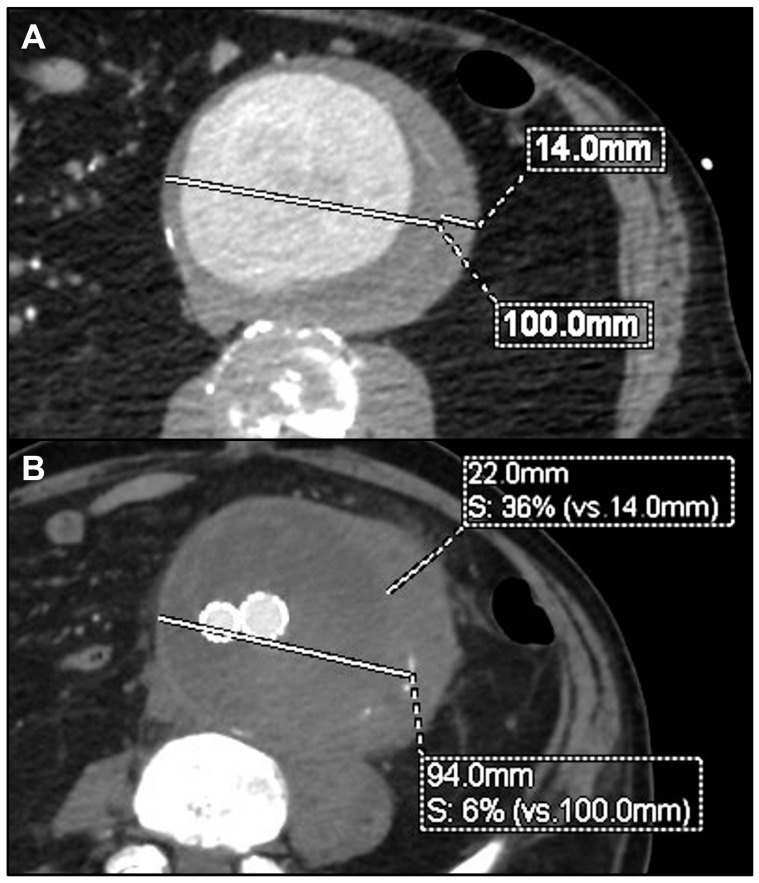
Preoperative computed tomography angiography (CTA) of symptomatic rapidly growing 100 mm infrarenal aneurysm (**A**) and 3-month postoperative CTA showing aneurysm’s shrinkage but significant growth of the peri-aortic inflammatory tissue (**B**).

During the mean follow-up of 31.6 ± 23.1 months (range 1–71), no further death or major adverse event was registered among the remaining 13 alive patients. Re-interventions were performed in three cases (20%). One patient originally treated with TEVAR required, 1 month later, a proximal thoracic extension and left subclavian artery coverage and embolization for disease progression. Another patient treated with BEVAR underwent, after 1 year, TEVAR for a new-onset isolated non-contiguous aortic arch saccular lesion. The third patient, previously treated with a straight abdominal tube graft, required a distal extension with a bifurcated stent graft, after 5 months, for distal disease progression and rupture of the aortic bifurcation. The same patient presented an aneurysmal degeneration of the proximal aortic neck, which is currently under surveillance after 55 months from the index procedure (Figure 1). Another patient, treated with EVAR for symptomatic rapidly growing infrarenal aneurysm, showed after 3 months a significant growth of the peri-aortic inflammatory tissue, without an indication for a re-intervention so far (Figure 2). Except these, no other patient reported any signs of recurrences or endograft infection during the follow-up. No case of open conversion was registered. Aneurysm shrinkage > 5 mm or stability was noted in 10 of the 13 patients who survived the early period after repair. Preoperative and postoperative details for each patient are summarized in Table 2.

## 4. Discussion

The management of infective aortitis remains one of the most challenging problems for the vascular surgeon, due to its rarity and the difficulty in choosing the best therapeutic option. Infective aortitis is frequently associated with complicating factors, such as rupture, sepsis, and suprarenal location, potentially resulting in increased morbidity and mortality [6,7]. The initial treatment in the case of suspected infective aortitis is intravenous antibiotic therapy with broad antimicrobial coverage of the most likely pathologic organisms. Antibiotics should be initiated as soon as the diagnosis of infective aortitis is suspected, and while awaiting microbiologic data. The antibiotic regimen can then be tailored, based upon the results of the blood culture [8,9,10]. Due to case series reporting a very high mortality rate among patients with infective aortitis treated with medical therapy alone, a combination strategy of intensive antibiotic therapy and surgical repair is generally recommended, although no clinical trials have explored the optimal management of such patients [11,12,13].

Despite the lack of evidence, open surgery continues to be considered the standard of care for the definitive treatment of infective aortitis. Conventional open repair allows resection of the aneurysm, local debridement, and in situ or extra-anatomic reconstructions. Options for in situ conduits include autologous or heterologous cryopreserved femoral arteries or veins, bovine pericardium, or prosthetic grafts in the case of unavailability [14].

**Figure 3:**
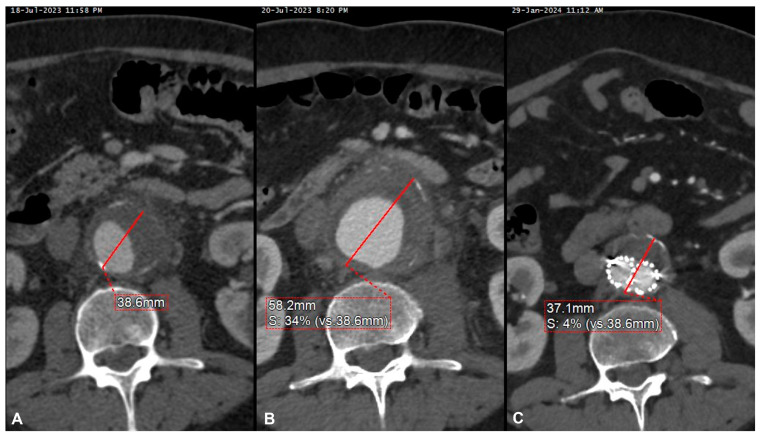
Pre-operative computed tomography angiography (CTA) of symptomatic small abdominal aortic aneurysm (**A**) and 2-day CTA performed for persistent pain showing a 20 mm aneurysm’s growth (**B**), urgently treated with EVAR with a good clinical and radiological result, as demonstrated by the significant aneurysm’s shrinkage noted at the 6-month postoperative CTA (**C**).

Although the clinical presentation may widely vary, the most common presentation in our sample was a rapidly growing aneurysm, with symptoms of abdominal or back pain, with evidence of contained or free rupture in half of the cases. This required a prompt interventional management in all of our patients, in addition to antibiotic therapy (Figure 3).

Open surgery is generally the standard of treatment for aortic aneurysms associated with aortitis, although endovascular techniques have recently been successfully employed [15,16,17]. However, very few comparative studies have been conducted so far to identify the best management between open and endovascular techniques for patients with aortitis. Furthermore, results from these experiences are highly conditioned by a selection bias based on clinical and radiological findings, since patients with worse presentation are generally managed by open surgical repair, and inevitably this affects the outcomes [18,19].

Oderich et al. reported one of largest observations of open repair of infective aortitis, including 42 patients managed with in situ aortic reconstruction or extra-anatomic bypasses over a 25-year period. The authors reported an operative mortality of 21% and a cumulative survival rate at 1 and 5 years of 82% and 50%, respectively, significantly lower than survival rates for the general population and for the non-infected aortic aneurysm. The authors found that for extensive periaortic infection, mostly sustained by Staphylococcus aureus, female sex, aneurysm rupture, and suprarenal aneurysm location were all variables associated with increased risk of aneurysm-related death (*p* < 0.05) [20]. The open approach implies surgical resection, soft-tissue debridement, and remote arterial reconstruction out of the field of infection. However, this kind of treatment cannot be considered risk-free for what concerns the tightness of aortic sutures as well as the morbidity potentially associated with a major aortic repair. In addition, aneurysms located in the thoracoabdominal and paravisceral aorta are not amenable to conventional extra-anatomic reconstruction. In such cases, despite the prohibitive risk profile of these kinds of patients who are defined as in poor general condition, an in situ aortic replacement is often necessary. On the other hand, endovascular treatment of thoracoabdominal or paravisceral aneurysm is associated with reduced postoperative mortality and major morbidity if compared to open surgery [21,22], and in this specific setting has the theoretical advantage of avoiding extensive manipulation of inflamed aortic tissue, with subsequent reduced risk of infection dissemination and surgical graft-related complications. In our experience, 7 of the 15 patients (47%) presented with thoracic, thoracoabdominal, or paravisceral localization, requiring TEVAR, off-the-shelf BEVAR, or Ch-EVAR. Among these, only one 89-year-old patient died perioperatively because of multi-organ failure. Other two patients in this subgroup required a secondary procedure during the follow-up: one TEVAR for proximal disease progression, and another TEVAR for a new-onset isolated non-contiguous arch lesion.

**Figure 4:**
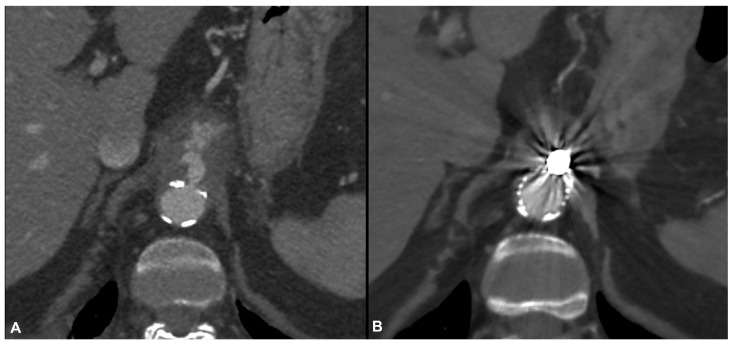
Pre-operative computed tomography angiography (CTA) of a contained ruptured supraceliac atheromatous plaque in a porcelain aorta (**A**) treated in emergency by TEVAR and occlusion of the celiac trunk by coils with good postoperative results as demonstrated by the postoperative CTA (**B**).

Since, during last decades, endovascular techniques for the treatment of aortic disease have been dramatically evolving for all anatomic locations, in many centers the endovascular option is today taken into account in the management of most cases, especially when faced with challenging repair such as in case of infective aortitis affecting the thoracic or suprarenal aorta. Mehra et al. reported a case of a thoracic pseudoaneurysm caused by a paravertebral abscess secondary to Tubercular involvement of the thoracic aorta successfully treated with TEVAR and antitubercular antibiotics [23]. Pulimamidi et al. described two cases of Salmonella aortitis successfully treated with EVAR, and provided a review of 27 cases over an 11-year period with a survival of 85% at 19-month follow-up [24]. Chen et al. described a case of aortitis secondary to Brucellosis causing rupture of the aorta at the level of the celiac trunk. The patient was successfully treated with TEVAR and long-term antibiotics [25]. This last patient showed an aortic lesion similar to that of the only female included in our sample, diagnosed with endocarditis and presenting with fever associated with abdominal and back pain, not responsive to medical therapy. The CTA showed a porcelain aorta and a leakage at the level of the ostium of the celiac trunk. The patient was treated in emergency by TEVAR and occlusion of the celiac trunk by coils, with immediate symptom regression (Figure 4). Interestingly, pain regression was achieved in all of our symptomatic patients just after the endovascular repair, confirming the hypothesis that the pain can derive more from the aortic lesion than from the inflammatory infiltrate. Similarly, Koeppel et al. reported a case of painful aortitis with positive blood cultures for Salmonella enteritidis, initially treated with pain relief therapy through peridural catheter without success. The same patient underwent endovascular treatment with subsequent immediate pain regression [26].

Three of our patients (20%) experienced disease progression during the follow-up. Two of these underwent a secondary procedure, of which one is currently under surveillance for a new recurrency. In addition, another patient required a re-intervention for a non-contiguous new lesion. This underlines the importance of a strict CTA imaging follow-up with the purpose of evaluating the residual sac and inflammatory infiltrate behavior, the integrity of proximal and distal landing zones, and possible development of new lesions in remote fields. Radiological findings should always be integrated and correlated with clinical and laboratory data, for a possible cyclical resumption of antibiotic therapy during the postoperative follow-up.

The three patients who underwent a secondary procedure contributed to a reintervention rate of 20% at the mean follow-up of 31.6 months. Although, according to our experience, the risk of reintervention remains relatively high after endovascular repair of infective aortitis, this should be interpreted in relation with the extension of the disease and the complexity of the procedures, which actually included three TEVAR, one chimney/periscope TEVAR, three BEVAR, and one chimney EVAR. However, the risk of mortality and major morbidity possibly associated with open surgery for such extensive pathologies would have remained substantial. In addition, the urgent or emergent clinical presentation of our patients justified the use of endovascular techniques, which can possibly represent a bridging therapy.

Although infective aortitis has always been considered a rare disease, in our two-center observation we found 15 confirmed cases over 5 years, and this may potentially represent an important finding of our research. The reason for such a surge in cases can be explained by the high sensitivity of modern diagnostic techniques and the increased chance of treatment, which today is not denied even to old and fragile patients, also thanks to the endovascular revolution.

Based on such evidence, the advantages of treating patients with infective aortitis by endovascular means remain substantial. Therefore, from the perspective of natural evolution towards mini-invasiveness in any surgical field, and in the light of the degree of maturity reached by endovascular techniques, it seems today even more reasonable to offer endovascular repair to these kinds of patients.

### Study Limitations

This was a retrospective observational analysis of prospectively collected data; therefore, the risk of selection and reporting bias is likely to be substantial. Additionally, the small sample size can lead to biased results, overestimation of effects, and low replicability.

## 5. Conclusions

Despite the relative reluctance to use an endograft in an infected area, in our experience the endovascular approach resulted to be feasible, safe, and effective in the treatment of infective aortitis with acute presentation, with acceptable peri-operative and mid-term follow-up outcomes. Our study has the limitation of being a relatively short observation of a small number of patients and the retrospective design has the risk of selection bias. Further studies are needed to confirm our results.

## Figures and Tables

**Table 1:** Baseline characteristics.

**Patients**	**15 (100%)**
Mean age (years)	74.2 ± 8.3 (range 61–87)
Male	14 (93%)
Female	1 (7%)
Hypertension	15 (100%)
Chronic kidney disease	6 (40%)
Chronic obstructive pulmonary disease	9 (60%)
Coronary artery disease	8 (53%)
Diabetes mellitus	7 (47%)
Mean aortic wall thickening (mm)	30.3 ± 19.2
**Organism isolated**	
Staphylococus Aureus	7 (47%)
*Salmonella* spp. Non-Typhi	3 (20%)
*Escherichia coli*	3 (20%)
*Brucella* spp.	1 (7%)
*Pseudomonas* and *Klebsiella*	1 (7%)
**Lesion location**	
Thoracic aorta	2 (13%)
Toracoabdominal and pararenal aorta	5 (33%)
Abdominal aorta	8 (53%)
**Endovascular technique**	
TEVAR	4 (27%)
BEVAR	3 (20%)
Ch-EVAR	1 (7%)
EVAR	7 (47%)

TEVAR: thoracic endovascular aneurysm repair; BEVAR: branched endovascular aneurysm repair; Ch-EVAR: chimney endovascular aneurysm repair; EVAR: endovascular aneurysm repair.

**Table 2:** Patients’ preoperative and postoperative details.

N	Sex	Age	Etiology	Organism Isolated	Fever	Lesion Characteristics	Antibiotic Therapy	Endovascular Technique	Outcome	FU (m)	FU Findings	Reintervention
1	M	85	Endocarditis	*Staphylococcus aureus*	Yes	Symptomatic rapidly growing thoracic PAU	Oxacyllin	TEVAR	Alive	48	Proximal disease progression	Redo TEVAR ± LSA embolization
2	M	70	Spondylodiscitis	*Staphylococcus aureus*	No	Contained ruptured type I TAAA	Linezolid	TEVAR ± SMA Chimney ± RRA Periscope ± CT embolization	Alive	52	Stability with type II endoleak	-
3	M	63	Spondylodiscitis	*Staphylococcus aureus*	No	Contained ruptured infrarenal atheromatous plaque ± periaortic abscess	Oxacyllin	EVAR	Alive	36	Stability	-
4	M	62	Endocarditis	*Staphylococcus aureus*	Yes	Contained ruptured PAAA	Linezolid ± Lamivudina	BEVAR	Alive	50	Shrinkage	-
5	M	68	Sepsis in porcelain aorta	*Salmonella* spp. Non-Typhi	Yes	Contained ruptured infrarenal atheromatous plaque	Ampicillin ± Sulbactam	EVAR (tube graft)	Alive	57	Proximal and distal disease progression	Bifurcated EVAR
6	F	75	Endocarditis	*Staphylococcus aureus*	Yes	Contained ruptured supraceliac atheromatous plaque	Oxacyllin	TEVAR ± CT embolization	Alive	20	Shrinkage	-
7	M	78	Spondylodiscitis	*Staphylococcus aureus*	No	Symptomatic multi-lobular type IV TAAA	Oxacyllin	BEVAR	Alive	36	Stability	-
8	M	72	Sepsis in pre-existing TAAA	*Escherichia coli*	Yes	Symptomatic saccular type IV TAAA ± peri-aortic gas	Amoxicillin ± Clavulanate	BEVAR	Alive	11	Stability ± non-contiguous new arch lesion	TEVAR
9	M	69	Sepsis in pre-existing AAA	*Salmonella* spp. Non-Typhi	Yes	Symptomatic rapidly growing AAA	Cotrimoxazol	EVAR	Alive	7	Increase in wall thickening	-
10	M	89	Pneumonia	*Pseudomonas* ± *Klebsiella*	Yes	Contained ruptured saccular TAA	Piperacillin ± Tazobactam	TEVAR	Death in 15° POD	-	-	-
11	M	87	Pyelonephritis	*Escherichia coli*	No	Symptomatic rapidly growing AAA	Piperacillin ± Tazobactam	EVAR	Alive	4	Stability	-
12	M	73	Pyelonephritis	*Escherichia coli*	No	Ruptured rapidly growing AAA	Piperacillin ± Tazobactam	EVAR	Death in 11° POD	-	-	-
13	M	75	Pancreatitis	*Brucella* spp.	No	Ruptured juxtarenal pseudoaneurysm	Doxycycline	Ch-EVAR	Alive	60	Stability	-
14	M	87	Pancreatitis	*Salmonella* spp. Non-Typhi	No	Symptomatic multiple infrarenal PAU	Cotrimoxazol	EVAR	Alive	16	Stability	-
15	M	61	Endocarditis	*Staphylococcus aureus*	No	Symptomatic rapidly growing AAA	Oxacyllin	EVAR	Alive	11	Shrinkage	-

FU: follow-up; PAU: penetrating aortic ulcer; TEVAR: thoracic endovascular aneurysm repair; LSA: left subclavian artery; TAAA: thoracoabdominal aortic aneurysm; EVAR: endovascular aneurysm repair; PAAA: pararenal aortic aneurysm; BEVAR: branched endovascular aneurysm repair; SMA: superior mesenteric artery; RRA: right renal artery; CT: celiac trunk; TAA: thoracic aortic aneurysm; POD: postoperative day; Ch-EVAR: chimney endovascular aneurysm repair.

## Data Availability

The original contributions presented in the study are included in the article, further inquiries can be directed to the corresponding author.
